# Differential expression of *var* subgroups and *PfSir2a* genes in afebrile *Plasmodium falciparum* malaria: a matched case–control study

**DOI:** 10.1186/s12936-019-2963-z

**Published:** 2019-09-23

**Authors:** Himanshu Gupta, Beatriz Galatas, Gloria Matambisso, Lidia Nhamussua, Pau Cisteró, Quique Bassat, Aina Casellas, Eusébio Macete, John J. Aponte, Charfudin Sacoor, Pedro Alonso, Francisco Saúte, Caterina Guinovart, Pedro Aide, Alfredo Mayor

**Affiliations:** 1 ISGlobal, Hospital Clínic - Universitat de Barcelona, Barcelona, Spain; 2 Centro de Investigação em Saúde de Manhiça, Manhiça, Mozambique; 3 ICREA, Pg. Lluís Companys 23, 08010 Barcelona, Spain; 4 Pediatrics department, Hospital Sant Joan de Déu (University of Barcelona), Barcelona, Spain; 5 Consorcio de Investigación Biomédica en Red de Epidemiología y Salud Pública (CIBERESP), Madrid, Spain; 6 National Institute of Health, Ministry of Health, Maputo, Mozambique; 7 Carrer Rosselló 153 (CEK Building), 08036 Barcelona, Spain

**Keywords:** Afebrile malaria, *var* genes, Gametocytes, *PfSir2a*, Mozambique

## Abstract

**Background:**

Poor knowledge on the afebrile *Plasmodium falciparum* biology limits elimination approaches to target asymptomatic malaria. Therefore, the association of parasite factors involved in cytoadhesion, parasite multiplication and gametocyte maturation with afebrile malaria was assessed.

**Methods:**

*Plasmodium falciparum* isolates were collected from febrile (axillary temperature ≥ 37.5 °C or a reported fever in the previous 24 h) and afebrile (fever neither at the visit nor in the previous 24 h) individuals residing in Southern Mozambique. *var*, *PfSir2a* and *Pfs25* transcript levels were determined by reverse transcriptase quantitative PCRs (RT-qPCRs) and compared among 61 pairs of isolates matched by parasite density, age and year of sample collection.

**Results:**

The level of *varC* and *PfSir2a* transcripts was higher in *P. falciparum* isolates from afebrile individuals (P ≤ 0.006), while *varB* and *DC8* genes (P ≤ 0.002) were higher in isolates from individuals with febrile infections. After adjusting the analysis by area of residence, doubling the relative transcript unit (RTU) of *varC* and *PfSir2a* was associated with a 29.7 (95% CI 4.6–192.3) and 8.5 (95% CI 1.9–32.2) fold increases, respectively, of the odds of being afebrile. In contrast, doubling the RTU of *varB* and *DC8* was associated with a 0.8 (95% CI 0.05–0.6) and 0.2 (95% CI 0.04–0.6) fold changes, respectively, of the odds of being afebrile. No significant differences were found for *Pfs25* transcript levels in *P. falciparum* isolates from afebrile and febrile individuals.

**Conclusions:**

*var* and gametocyte-specific transcript patterns in febrile and afebrile infections from southern Mozambique matched by age, parasite density and recruitment period suggest similar transmissibility but differential expression of variant antigens involved in cytoadhesion and immune-evasion.

## Background

Asymptomatic *Plasmodium falciparum* infections debilitate the health of affected population while representing a hidden source of continuous transmission that can compromise elimination efforts [1]. Poor knowledge on the dynamics, transmissibility and biological mechanisms of afebrile infections restrict the evidence-based approaches to deal with this hidden reservoir.

Transmissibility and clinical impact of *P. falciparum* infections is dependent on gametocyte production and parasite virulence [1]. Higher carriage of gametocyte stages has been observed among afebrile infections compared to febrile cases [2, 3]. The histone-modifying *Plasmodium* homologue of Sir2 (PfSir2a), a transcriptional factor that regulates ribosomal DNA transcription and *P. falciparum* multiplication rate [4], was found to be more expressed in severe malaria than in uncomplicated infections [5]. Moreover, *PfSir2a* has been implicated in the epigenetic regulation of *var* gene expression [6]. This multicopy gene family codes for the *P. falciparum* erythrocyte membrane protein 1 (PfEMP1) that mediates cytoadhesion of infected erythrocytes to host receptors. *var* genes can be subdivided as per the motifs present in non-coding sequences and locus position (groups A, B and C) and on the combination of domain cassettes (DCs) [7]. *var* groups A, B, as well as *DC*8, 11 and 13 have been associated with severe malaria [7, 8]. Conflicting results have been reported for the associations of *var* gene expression and asymptomatic malaria [9–11], probably due to marked differences in age and parasite densities among febrile and afebrile infections.

Parasite survival, virulence and transmission depend on a balanced investment in within-host replication and between-host transmission [12]. It is hypothesized that the decrease of parasite virulence and increased transmissibility would maximize the long-term persistence of afebrile infections in the human host environment. To test this, parasite transcription of genes involved in cytoadhesion, proliferation and gametocyte maturation were compared between parasites collected from Mozambican individuals with febrile and afebrile outcomes matched by age, parasite density and period of sample collection.

## Methods

### Study site and population

This study took place in the district of Manhiça (southern Mozambique) where community-based age-stratified cross-sectional surveys were conducted in May of 2012 to 2015. After giving written informed consent, participants were finger-pricked and their blood was used to detect *P. falciparum* infection by rapid diagnostic test (RDT), microscopy, quantitate real-time PCR (qPCR) and storage in RNAprotect. *Plasmodium falciparum*-infected individuals were defined as afebrile if their axillary temperature at the time of visit was < 37.5 °C without reporting having had fever in the preceding 24 h, and febrile if the axillary temperature was ≥ 37.5 °C at the time of visit or reported fever during the preceding 24 h. Febrile and afebrile cases were individually matched based on year of sample collection, age (0 to ≤ 5; > 5 to ≤ 15; > 15 to ≤ 25 and > 25 years) and parasite density (≤ 200, > 200 to ≤ 1000, and > 1000 parasites/µL). The Hospital Clínic (Barcelona, Spain) ethics review committee and National Mozambican Ethical Review Committee (Mozambique) approved the study.

### Parasitological determinations

Approximately 10 μL of finger-pricked blood were used to perform PfHRP2-based rapid diagnostic tests (SD BIOLINE Malaria Antigen P.f—05FK50) by trained technicians following the manufacturer’s instructions. Thin and thick blood smears were stained with Giemsa as described previously [13]. Two experienced microscopists independently read all slides, and a third reading was performed if there were discrepancies between the results of the first two. *Plasmodium falciparum* infections were also identified through qPCR from 25 μL of dried blood spots collected on filter papers. DNA was extracted using the QIAamp DNA Mini kit (Qiagen, Hilden, Germany), as per the manufacturer’s instructions. Parasite DNA amplification targeting the 18S rRNA gene was performed using the ABI PRISM 7500 HT Real-Time System (Applied Biosystems, Foster City, USA), following a method described elsewhere [14, 15]. Then, a standard curve was prepared on the foundation of an in vitro culture of the 3D7 strain, which contained known numbers of ring-infected erythrocytes. The standard curve was performed for each test in triplicate with five serial dilutions. Using the 18S rRNA gene as an amplification target, parasitaemia in the clinical samples were quantified and then subjected to extrapolation against the standard curve [16].

### RNA extraction

Extraction of RNA from *P. falciparum* infected blood (50 µL) preserved in 250 µL RNAprotect (Qiagen, Hilden, Germany) was performed using 700 µL of Trizol (Ambion, Life Technology, California, USA) following manual extraction with phenol/chloroform phase separation to elute total RNA. Extracted total RNA was subjected to DNase treatment using DNase Max kit (Qiagen, Hilden, Germany) at 37 °C for 30 min followed by reverse transcription of 400–800 ng of total RNA using PrimeScript™ RT Master Mix reagents (Takara, Shiga, Japan). To confirm the removal of gDNA or the synthesis of cDNA, qPCR was performed using primers targeting *P. falciparum* ubiquitin-conjugating enzyme (*PF08_0085*) on reverse transcription positive and negative controls (RNA samples without reverse transcriptase enzyme).

### Quantification of transcript levels

Transcript levels of *var* genes such as *varA*-exon2, *varA*-DBLα1 (*varA*-*notDC3*), *varB* group (*varB*-UTR region) and *varC* group (*varC*-UTR region) were assessed using degenerate primers and quantitative reverse transcriptase PCRs (RT-qPCRs) [7, 10, 17]. Domain cassette transcript levels were also determined using a set of primers targeting semi-conserved domains belonging to *DC8* (CIDRα1.1), *DC11* (CIDRβ2 + DBLγ7) and *DC13* (CIDRα1.4) [7, 8]. The assessment of transcript level of genes involved in parasite multiplication (*PfSir2a*) and gametocyte mature stage (*Pfs25*) was also performed using previously described RT-qPCR primers [4, 18, 19]. *PF08_0085* gene was used as a housekeeping gene [20]. In brief, 96-well plates containing individual 20 μL reactions were amplified in triplicates using a 7500 HT Real-Time System (Applied Biosystem, Foster City, USA). Each reaction mixture had 10 μL of 2X Power SYBR^®^ Green PCR Master Mix (Thermo Fisher Scientific, Warrington, WA, UK), 1 or 0.33 μM of each forward and reverse primers and 5 μL of template cDNA. The reaction volume was prepared with PCR-grade water. Amplifications were performed with a holding for 3 min at 50 °C, initial denaturation for 10 min at 95 °C, followed by 40 cycles of 95 °C for 15 s and 60 °C or 55 °C for 1 min. Samples with a Ct > 40 for any gene target were considered as not expressed. The specificity of primer pairs against human gDNA was also determined. The 7500 System SDS software v1.4 was used to analyse the collected data. A standard curve was then used to calculate the PCR efficiency of each primer from 7 log dilutions of the *P. falciparum* 3D7 gDNA, by using the formula (E = 10^−1/m^), where “*m”* was the slope. The formula *C/E*^Δ*Ct*^ was used to convert Ct values to copy numbers [9]. In the formula, “*C”* was the number of copies of the gene in the *P. falciparum* 3D7 genome, “*E”* was the efficiency of the PCR [7, 9] and ΔCt was the difference in Ct values between a sample and *P. falciparum* 3D7 reference gDNA loaded in each plate [21]. The relative transcript unit (RTU) of target genes was calculated by dividing the target gene transcript levels by *PF08_0085* (housekeeping gene) transcript levels. Non-template controls were tested in every plate [8].

### Statistical analysis

Wilcoxon matched-pairs signed rank test and McNemar’s Chi squared test were used to compare continuous and categorical variables, respectively. The association between being febrile or afebrile and log-transformed RTUs was tested by conditional logistic regression models adjusted by area of residence in order to account for differences in transmission intensities between areas. All statistical analyses were performed using R 3.3.2, and GraphPad Prism 5.0 (GraphPad Software) was used to generate graphs.

## Results

**Fig. 1:**
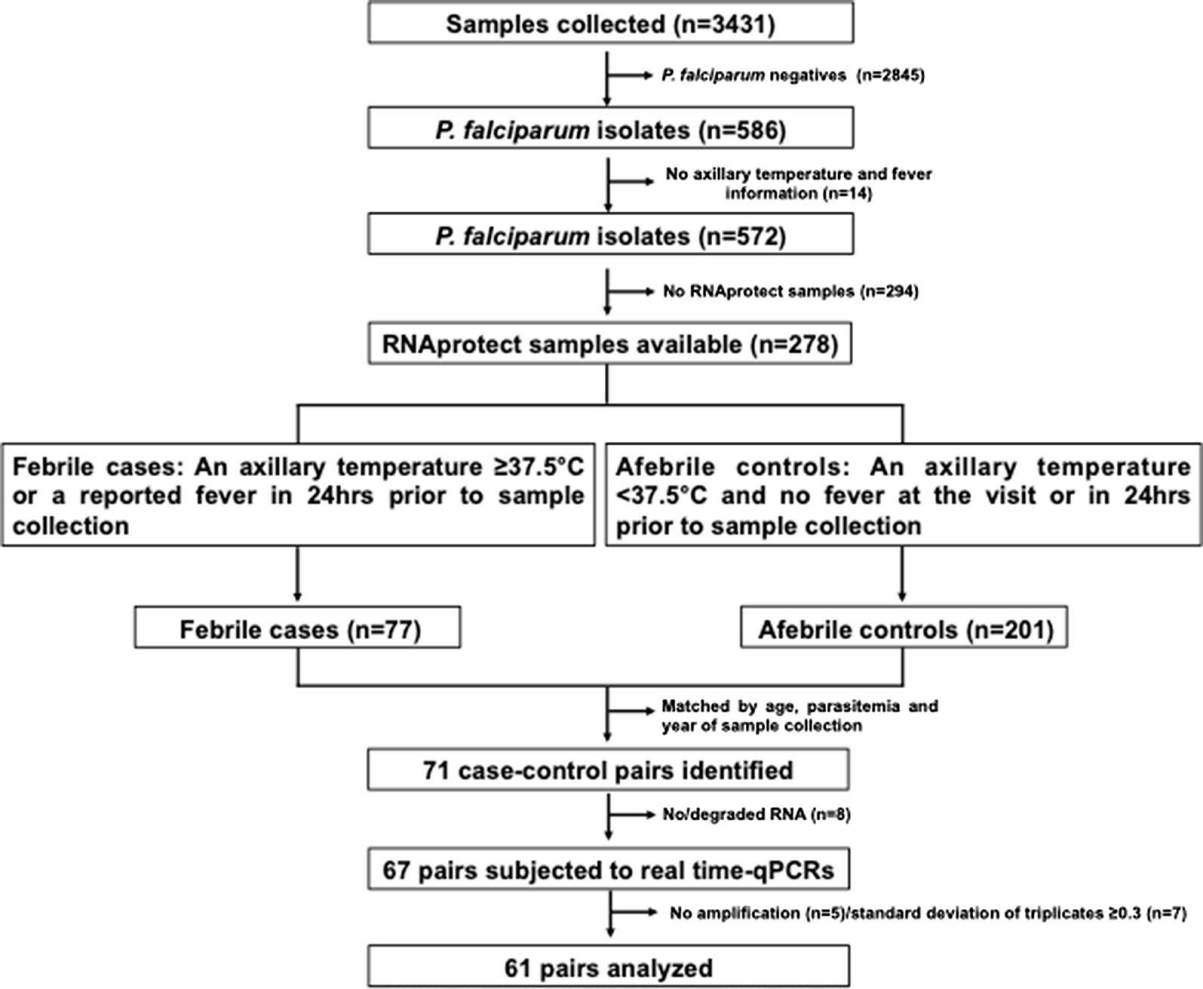
Schematic representation of sample selection for reverse transcriptase quantitative PCR analysis

Among the 3431 participants in cross-sectional surveys from whom blood samples were collected, 278 were *P. falciparum* positive by qPCR, microscopy and/or RDTs, had information on axillary temperature as well as on fever in the previous 24 h, and had available blood samples in RNAprotect (Fig. 1). Two hundred and one of them (72.3%) were afebrile. Seventy-one of them were matched with febrile individuals with similar age, parasite density and year of sample collection.

The transcript analysis of target genes was successful in 61 (86.0%) of the 71 pairs (Fig. 1). Thirteen (21.3%) of the febrile individuals had a fever at the time of recruitment and 48 (78.7%) reported having a fever in the preceding 24 h (but had no fever at recruitment). No differences in median age (4.7 years, 95% confidence interval [CI] 2.1–15.2 in afebrile individuals and 5.0 years, 95% CI 3.0–15.1 in febrile individuals, P = 0.979), parasite densities (542.6 parasites/μL of blood, 95% CI 59.7–4267.2 in afebrile individuals and 649.4, 95% CI 45.4–5897.7 in febrile individuals, P = 0.764), sex (P = 0.377) and area of residence (Maragra, Manhiça, Malavele, Palmeria, Taninga and IIha Joshina Machel; P = 0.812) were observed among febrile and afebrile individuals (Table 1).

**Table 1:** Main characteristics of matched case–control study participants, 2012–2015

Variable	Afebrile (N = 61)	Febrile (N = 61)	P value
Year, n(%)			
2012	4 (6.5)	4 (6.5)	NA
2013	20 (32.8)	20 (32.8)	
2014	22 (36.1)	22 (36.1)	
2015	15 (24.6)	15 (24.6)	
Age, years, n(%)			
0 ≤ 5	33 (54.1)	33 (54.1)	NA
5 ≤ 15	13 (21.3)	13 (21.3)	
15 ≤ 25	2 (3.3)	2 (3.3)	
> 25	13 (21.3)	13 (21.3)	
Parasite density (parasites/µL, n(%))			
0– ≤ 200	24 (39.4)	24 (39.4)	NA
> 200– ≤ 1000	11 (18.0)	11 (18.0)	
> 1000	26 (42.6)	26 (42.6)	
^a^Axillary temperature, n(%)			
≥ 37.5 °C	0 (0)	13 (21.3)	< 0.001
< 37.5 °C	61 (100)	48 (78.7)	
Reported fever in the previous 24 h, n(%)			
Yes	0 (0)	58 (95.1)	< 0.001
No	61 (100)	3 (4.9)	
Sex, n(%)			
Male	31 (50.8)	25 (41.0)	0.377
Female	30 (49.2)	36 (59.0)	
^b^Area, n(%)			
Maragra	4 (6.5)	10 (16.4)	0.812
Manhiça	13 (21.3)	10 (16.4)	
Malavele	0 (0)	5 (8.2)	
Palmeira	22 (36.1)	17 (27.9)	
Taninga	3 (4.9)	3 (4.9)	
Ilha Josina	19 (31.2)	16 (26.2)	

McNemar’s Chi squared test—categorical data; *NA* Not applicable

^a^at the time of visit

^b^Conditional logistic regression controlling for the matched pairs

**Fig. 2:**
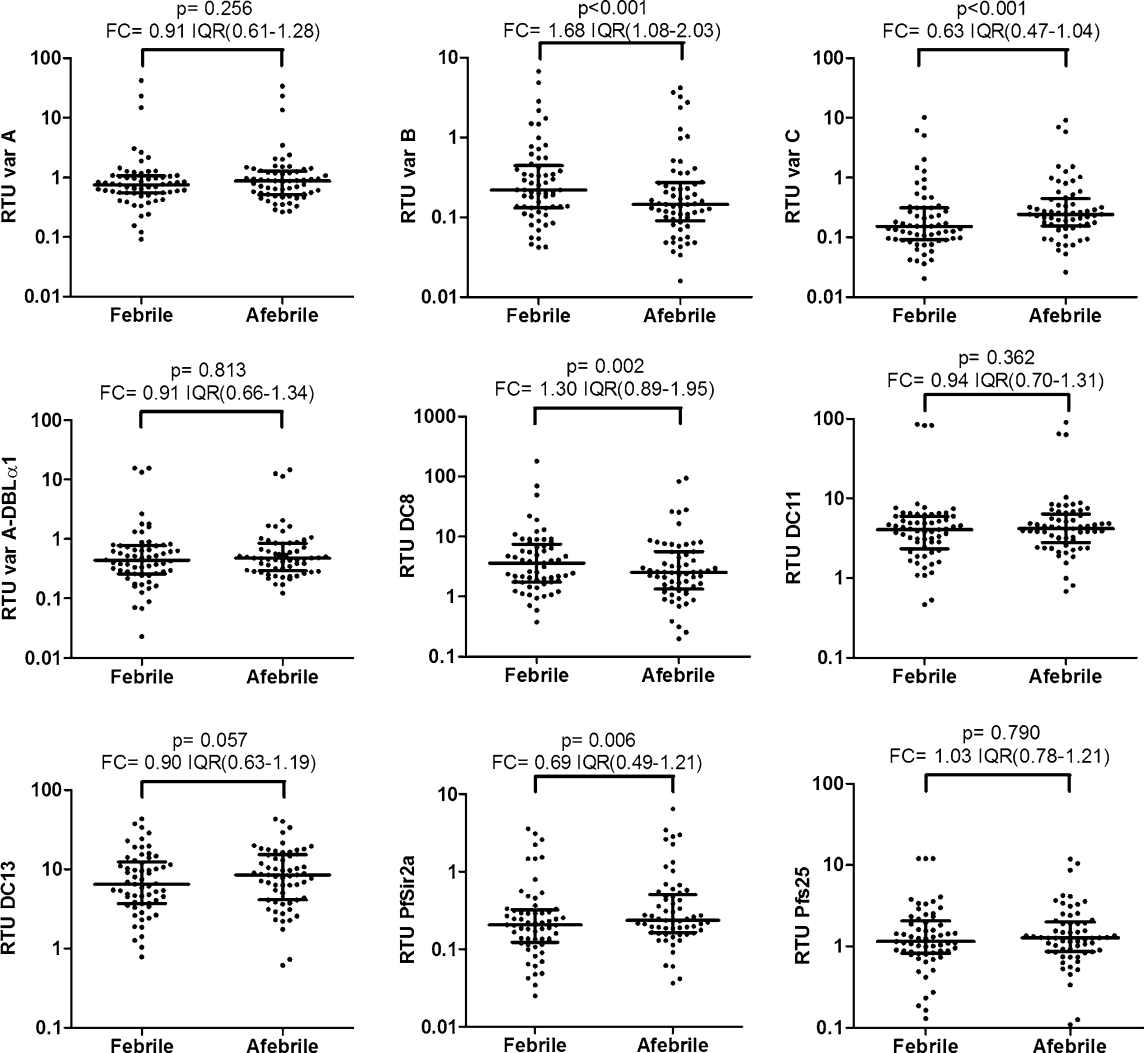
*Plasmodium falciparum* transcript levels in febrile and afebrile infections. Relative transcript units (RTUs) were calculated with respect to the *PF08_0085* housekeeping gene in *P. falciparum* infections from individuals with fever (axillary temperature ≥ 37.5 °C or reported fever during the preceding 24 h) and afebrile individuals (axillary temperature < 37.5 °C at recruitment) matched by year, age and parasite density. P values were obtained from Wilcoxon matched-pairs signed ranks test. T bars represent median and Interquartile Ranges (IQR). Overall fold change (FC) was calculated as the median and IQR of the RTU ratios between febrile and afebrile pairs

The RT-qPCR efficiencies of targeted genes ranged between 83.0% and 97.6% (Table 2). Among the 9 tested genes, RTUs of 4 of them (*varB*, *varC*, *DC8* and *PfSir2a*) were statistically different between afebrile and febrile infections (Fig. 2). The level of *varC* and *PfSir2a* transcripts was higher in *P. falciparum* isolates from afebrile individuals (P ≤ 0.006), while *varB* and *DC8* genes (P ≤ 0.002) were higher in isolates from individuals with febrile infections. After adjusting the analysis by area of residence, doubling the RTU of *varC* and *PfSir2a* was associated with a 29.7 (95% CI 4.6–192.3) and 8.5 (95% CI 1.9–32.2) fold increases, respectively, of the odds of being afebrile. In contrast, doubling the RTU of *varB* and *DC8* was associated with a 0.8 (95% CI 0.05–0.6) and 0.2 (95% CI 0.04–0.6) fold changes, respectively, of the odds of being afebrile.

**Table 2:** PCR efficiencies of each gene used for qPCR analysis

Gene	Gene copies in 3D7 genome	PCR efficiencies (%)
*varA*-exon2	10	89.6
*varA*-DBLα1	6	92.0
*varB*-UTR	22	88.3
*varC*-UTR	7	83.0
*DC8* (CIDRα1.1)	1	88.6
*DC11*	2	89.5
*DC13*	1	90.2
*Pfs25*	1	97.6
*PfSir2a*	1	92.7
*PF08_0085*	1	93.4

## Discussion

This study identified higher abundance of *var* group C and *PfSir2a* transcripts in afebrile *P. falciparum* infections compared to age- and parasite density-matched febrile infections, while opposite patterns were observed for *var* group B and *DC8* transcript levels. Similar gametocyte levels were observed in both types of infections, based on the lack of differences observed for *Pfs25* transcripts. Overall, these findings suggest that the expression of variant surface antigens involved in cytoadhesion and immune-evasion can determine the persistence of afebrile infections.

*var* group B and *DC8* genes found to be more expressed in febrile than afebrile infections have been previously linked to cytoadhesion phenotypes (i.e., endothelial protein C receptor) associated with severe malaria [7, 22], thus suggesting a higher pathogenic potential that could drive infections towards clinical manifestations. The higher level of *var* C transcripts in afebrile infections might indicate the presence of less-pathogenic variant surface antigens compatible with the subclinical persistence of these infections [9]. Increased immunity in afebrile individuals in spite of the matching by host age, or longer durations of infections, may have driven the exhaustion of the parasite *var* gene repertoire, leading to the expression of *var* group C variants capable of evading immunity though higher switch-off rates [1, 9]. The higher expression of *PfSir2a* in afebrile infections might have contributed to this switch towards *var* C subgroups [6, 23], suggesting the potential involvement of epigenetic mechanisms in afebrile malaria.

This study demonstrates a few noted discrepancies with previous results as well as several limitations. To begin, the association of *PfSir2a* with afebrile malaria is contradictory to a finding where *PfSir2a* was found associated with severe malaria [5]. The results of this study may have been influenced by the following limitations (a) febrile individuals identified in the community were not sick enough to seek for clinical care and may present with less pathogenic infections than infections in individuals who attend clinics, (b) uncomplicated and severe malaria patients could co-exist in the febrile group and (c) it is not possible to discard that afebrile infections at the time of the survey may progress to fever during subsequent days. Different criteria have been used to describe afebrile and febrile infections [24, 25]. However, several studies defined infections similarly to the present study [3, 10, 24–28]. Similar gametocyte levels were present in this study. However, higher carriage of gametocyte stages has been observed among afebrile infections at the time of recruitment compared to febrile cases in Tanzania and Burkina Faso [2, 3]. This discrepancy may have been due to matching parasite densities in febrile and afebrile groups of the present study. Moreover, it has been shown that the higher a person’s parasitaemia, the greater the chance of being gametocyte positive [29]. It has also been demonstrated that the detection of *Pfs25* transcripts was strongly dependent on parasite density [30]. Lastly, this study targeted a limited number of parasite genes given limited availability of RNA for transcriptional analysis, and may thus have missed some other parasite genes driving afebrile infections.

## Conclusion

Major differences exist in *var* and *PfSir2a* gene expression between parasites collected from Mozambican individuals with febrile and afebrile outcomes when matched by host age, parasite density and time-period of sample collection. Increased expression of *var C* genes may increase parasite persistence through a balance between antigenic escape and cytoadhesion to avoid splenic clearance. Several epigenetic mechanisms that include *PfSir2a* expression might contribute to this transcriptional shift [6]. In contrast, similar gametocyte-specific transcript levels in febrile and afebrile infections suggest that both types of infection are equally transmissible at same parasite density levels.

## Data Availability

The datasets analyzed in this study are available from the corresponding author on request.

## Abbreviations

PCR: polymerase chain reaction
RT-qPCRs: reverse transcriptase quantitative PCRs
RTU: relative transcript unit
PfSir2a: *Plasmodium* homologue of Sir2
PfEMP1: *Plasmodium falciparum* erythrocyte membrane protein 1
DCs: domain cassettes
RDT: rapid diagnostic test
qPCR: quantitate real-time PCR
IQR: interquartile ranges
FC: fold change
